# Soil Water Content Sensor Response to Organic Matter Content under Laboratory Conditions

**DOI:** 10.3390/s16081239

**Published:** 2016-08-05

**Authors:** Ali Fares, Ripendra Awal, Haimanote K. Bayabil

**Affiliations:** College of Agriculture and Human Sciences, Prairie View A&M University, Prairie View, TX 77446, USA; riawal@pvamu.edu (R.A.); hkbayabil@pvamu.edu (H.K.B.)

**Keywords:** capacitance sensor, 10HS sensor, organic matter, sensor precision, sensor accuracy

## Abstract

Studies show that the performance of soil water content monitoring (SWCM) sensors is affected by soil physical and chemical properties. However, the effect of organic matter on SWCM sensor responses remains less understood. Therefore, the objectives of this study are to (i) assess the effect of organic matter on the accuracy and precision of SWCM sensors using a commercially available soil water content monitoring sensor; and (ii) account for the organic matter effect on the sensor’s accuracy. Sand columns with seven rates of oven-dried sawdust (2%, 4%, 6%, 8%, 10%, 12% and 18% v/v, used as an organic matter amendment), thoroughly mixed with quartz sand, and a control without sawdust were prepared by packing quartz sand in two-liter glass containers. Sand was purposely chosen because of the absence of any organic matter or salinity, and also because sand has a relatively low cation exchange capacity that will not interfere with the treatment effect of the current work. Sensor readings (raw counts) were monitored at seven water content levels (0, 0.02, 0.04, 0.08, 0.12, 0.18, 0.24, and 0.30 cm^3^ cm^−3^) by uniformly adding the corresponding volumes of deionized water in addition to the oven-dry one. Sensor readings were significantly (*p* < 0.05) affected by the organic matter level and water content. Sensor readings were strongly correlated with the organic matter level (*R*^2^ = 0.92). In addition, the default calibration equation underestimated the water content readings at the lower water content range (<0.05 cm^3^ cm^−3^), while it overestimated the water content at the higher water content range (>0.05 cm^3^ cm^−3^). A new polynomial calibration equation that uses raw count and organic matter content as covariates improved the accuracy of the sensor (RMSE = 0.01 cm^3^ cm^−3^). Overall, findings of this study highlight the need to account for the effect of soil organic matter content to improve the accuracy and precision of the tested sensor under different soils and environmental conditions.

## 1. Introduction

Soil moisture measurements at different scales remain challenging due to complexities and often confounding edaphic and topographic factors. A study by [1] showed that the variability of soil water content at the field scale could be reasonably explained by texture and soil organic carbon (SOC) based on their study that relied on gravimetric soil moisture measurements and soil organic matter determination using the loss-on-ignition (LOI) method. While the gravimetric method of soil water content measurement is known for its accuracy, its approach is inefficient due to high costs in labor and time. 

Recent advancements in moisture measurement techniques that use soil electrical properties have overcome most of these challenges [2,3,4], and with the increasing use of in situ and remote sensing techniques (e.g., radiometer, thermal infrared techniques, microwave sensors), soil water content data are becoming readily available at relatively higher spatial and temporal resolutions [5,6]. The availability of such data is needed in different scientific disciplines, e.g., hydrology, ecology, crop production, and the area of natural resources management [7,8,9]. Moreover, continuous research on in situ sensing technologies has improved the performances and applications of soil water content sensors for different purposes and under various environments [10,11]. 

Despite the increased use of the soil water content monitoring (SWCM) sensors, however, several studies have shown that the accuracy and precision of these sensors are affected by soil physical properties, e.g., bulk density, compaction, porosity, and temperature [4,8,11,12,13,14] and media chemical properties, e.g., electrical conductivity and salinity [13,15,16,17]. Sensor precision does not always guarantee accuracy; sensor reading could be precise but at the same time inaccurate when sensor readings deviate from the actual values. A study by [8] found that readings of SWCM sensors were both negatively and positively correlated with the temperature depending on the soil saturation levels in sandy soils. Similarly, exaggerated water content readings were observed in saturated soil with saline water [18]. Several investigators [13,19,20] suggested that soil-specific calibrations of SWCM sensors are needed for better accuracy. 

While the effects of most of the soil properties on sensor performance are well documented, there is very little or almost no information on the effect of soil organic matter on the performance of SWCM sensors. A study by [5] reported that SOC significantly affected the soil moisture content at the field scale. In addition, it is well known that organic matter affects different soil physical and chemical parameters (e.g., bulk density, porosity, structure, and pH) [21,22,23,24]. Nevertheless, there are no published works on the effects of organic matter on the precision and accuracy of SWCM sensors. Understanding the effect of organic matter on soil moisture sensor readings will help the efforts to develop effective approaches to up-/down-scale moisture readings at different spatial and temporal scales. 

Therefore, the objectives of this study are to (i) investigate the effect of soil organic matter on the precision and accuracy of a commercially available, 10HS (Decagon Devices, Pullman, WA, USA) SWCM sensor; and (ii) mitigate the effect of organic matter on the SWCM sensor’s accuracy. Based on an extensive literature review, it is safe to say that this is the first scientific study that investigates the effect of organic matter on SWCM sensors. Results from this study will provide new insights on this topic and highlight the need to account for the soil organic matter effect on these sensors. 

## 2. Materials and Methods

### 2.1. Experimental Setup

Effects of organic matter level on the accuracy and precision of the commercially available 10HS soil water content monitoring sensor (Decagon Devices, Pullman, WA, USA) were studied under laboratory conditions. Sand columns were prepared by packing washed (using deionized water to remove any impurities) and oven dried quartz sand with mean diameter of 0.4 mm in 2 L (12 cm-diameter) glass beakers. According to Decagon Devices, idealized measurement volume of Decagon 10HS sensor is 10 cm diameter [25].

Sand was purposely chosen because it (i) is clean from any organic matter or salinity; (ii) does not shrink or swell; and also (iii) has a relatively low cation exchange capacity (CEC) that will not interfere with the treatment effect of the current work. Several capacitance sensor studies were also conducted using sand columns [8,16,26,27,28,29].

Moreover, sandy soils (with up to 96% pure sand) cover significant areas especially in arid and semi-arid regions throughout the world. These soils are characterized by their low moisture holding capacity and CEC. A slight change in its moisture status or organic matter content will have significant effects on their hydrology and the ecosystems they support. As a result, high precision and accuracy sensors are needed to quantify water content of sandy soils. Sawdust was used as the soil organic matter. Sawdust was chosen for its inert chemical properties to ensure no or a very limited effect on sensor readings. Sawdust, with particle sizes between 0.025 and 2 mm, was manually mixed with oven dried quartz sand before packing the mixture in the columns. Sand was mixed with the appropriate amounts of sawdust to achieve the eight organic content rates (0% (control), 2%, 4%, 6%, 8%, 10%, 12% and 18% v/v) used in this study. Each mixture was packed into a 2 L glass container; at corresponding bulk densities of 1.67, 1.63, 1.61, 1.59, 1.58, 1.54, 1.53 and 1.45 g cm^−3^, respectively. The appropriate volumes of deionized water were added during mixing to bring the water content to the desired water content levels (0.02, 0.04, 0.08 0.12, 0.18, 0.24, and 0.30 cm^3^ cm^−3^). No water was added to the 0 cm^3^ cm^−3^ water content treatment. Water content measurements were taken at room temperature (22 °C) using three 10HS SWCM sensors (Decagon Devices). The three sensors were placed into the sand columns for more than five minutes to ensure at least three repeated readings (raw counts) at each water content level per sensor. The experimental design was a factorial design with organic matter level, sensor type (which represents sensor to sensor variability), and water content as treatment variables. 

### 2.2. Correcting Organic Matter Effect on Sensor Readings

Raw count outputs of the sensors were converted into volumetric water content using the default calibration equation (Equation (1)) provided by the manufacturer [30]. According to the manufacturer, the sensor measures the soil water content within ±0.03 cm^3^ cm^−3^ [30].

*θ_p_* = (1.17 × 10^−9^ × RC^3^) − (3.95 × 10^−6^ × RC^2^) + (4.90 × 10^−3^ ×RC) − 1.9 (1)

where *θ_p_* is the apparent water content (cm^3^ cm^−3^), and RC is the raw count of the sensor. RC values depend on the air-water-soil mixture dielectric value of the medium; and thus, higher RC values indicate higher water content.

The default calibration equation provided by the manufacturer does not account for the effect of organic matter on sensor readings. Therefore, to account for the effect of soil organic matter level on sensor readings, organic-matter-level–specific and multivariate polynomial calibration equations were developed. The multivariate polynomial calibration equation was developed using the statistical package R [31] by incorporating raw counts and organic matter level (OM) as covariates. 

### 2.3. Statistical Analysis

An analysis of variance (ANOVA) test was performed using Statistix 10 software package (Analytical Software 2015, Tallahassee, FL, USA) to test the effect of organic matter level, sensor type (ST, which represents sensor to sensor variability), and water content (WC) on sensor readings (raw counts, RC) using a factorial experimental design. Statically significant interactions between two or more parameters required the use of the Tukey honest significance difference (HSD) mean separation techniques to separate the specific effect of each parameter. 

In addition, the mean absolute error (MAE) between apparent (*θ_p_*) and actual (*θ*) water contents was also calculated at each organic matter level (Equation (2)). The mean absolute error (MAE) was used to evaluate the accuracy of the default calibration equation in estimating the actual water content.

(2) $$\text{MAE}=\frac{\sum \left|\theta_{p\left(i\right)}-\theta_{\left(i\right)}\right|}{N}$$

where MAE is the mean absolute error, $\theta_{p}$ and *θ* are predicted and actual volumetric water contents (cm^3^ cm^−3^), respectively, and *N* is the number of observations.

## 3. Results and Discussion

### 3.1. Sensor Readings (Raw Counts)

The analysis of variance results of the sensor readings (raw counts) as a function of the organic matter level, sensor number, and water content are presented in Table 1. Overall, all three factors (OM, ST, and WC) and their interactions significantly affected (*p* < 0.05) sensor readings (Table 1).

**Figure 1:**
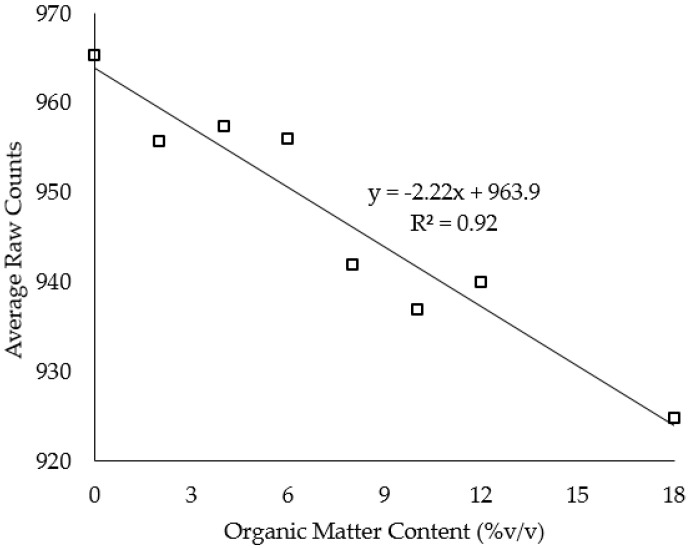
Correlation between organic matter level and average sensor readings (*n* = 72).

There is a strong negative correlation between the organic matter level and sensor readings (*R*^2^ = 0.92) (Figure 1). Thus, the greatest sensor readings were observed in the quartz sand without organic matter amendment (0% sawdust); as the organic content matter increased, sensor readings significantly decreased. The smallest average sensor readings were measured in soil columns with the highest (18% sawdust) organic matter level (Figure 1). 

### 3.2. Sensor Precision and Accuracy

**Figure 2:**
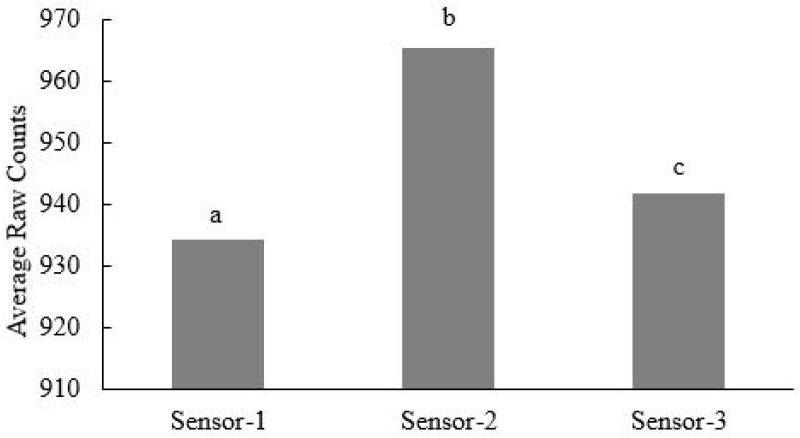
Sensor-to-sensor variation (Senor-1, Sensor-2, and Sensor-3) of sensor readings based on the results of the Tukey HSD test. Sensors not sharing the same letter are significantly different at *p* < 0.05.

Analysis of variance (Table 1) and post-hoc Tukey HSD mean comparison test results (Figure 2) demonstrate the significant sensor-to-sensor variation. Thus, readings from the three different sensor prototypes were significantly different (*p* < 0.05) (Figure 2). Contrary to the findings of [32], who reported small sensor-to-sensor variability of the 10HS sensor, our results show that the 10HS soil water content sensor is likely less precise.

**Figure 3:**
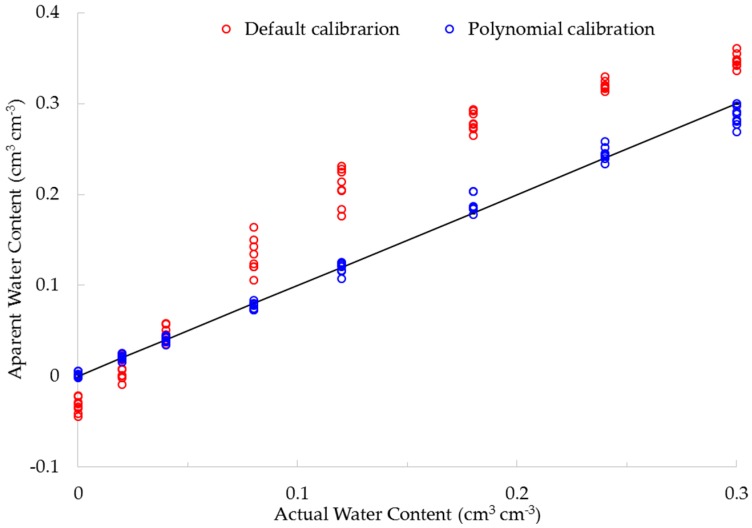
Scatter plot of the actual water content vs. apparent water content predicted using the default calibration equation.

These results concur with those of [33], who reported significant differences between the replication of ECH_2_O sensors, although the present sensor has a different design and working frequencies than the ECH_2_O sensor. In addition, the two-way interaction between the sensor type and organic matter level indicates a significant variation of the sensor readings (Figure 3). However, although Sensor-2 resulted in significantly greater sensor readings at a lower organic matter level (0% and 2%), overall there was no consistent trend in the interaction between the sensor type and organic matter level on the sensor readings (Figure 2).

**Figure 4:**
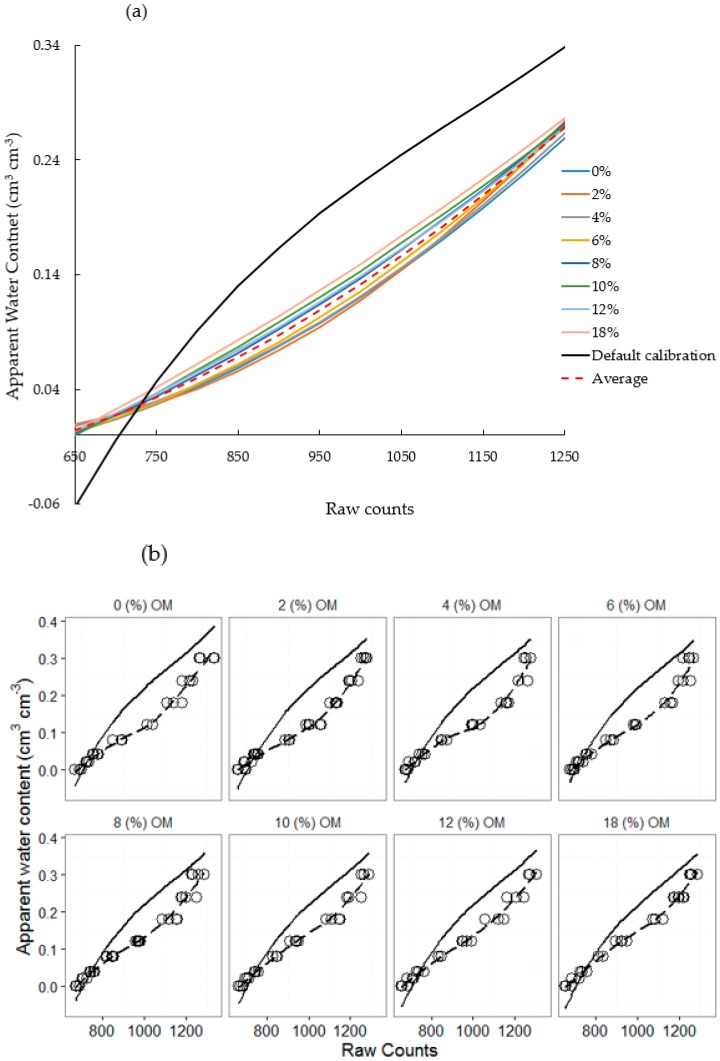
Actual (open circles) and apparent water contents (solid black lines) regressed against SWCM raw counts for different organic matter levels; (**a**) all plots combined and (**b**) separate plots for each organic matter level.

The default calibration underestimates the actual water content, and there are even negative readings (e.g., see the “red” circles in Figure 3) for the lower water content range (<0.05 cm^3^ cm^−3^). However, it considerably overestimated the actual water content at the higher water content range (>0.05 cm^3^ cm^−3^ e.g., see the “blue” circles in Figure 3). Similarly, at lower sensor ranges, the apparent water content (predicted using the default calibration equation) was lower than the actual water content; conversely, with the increase in sensor readings, the apparent water content appeared to be greater than the actual (Figure 4) water content.

**Figure 5:**
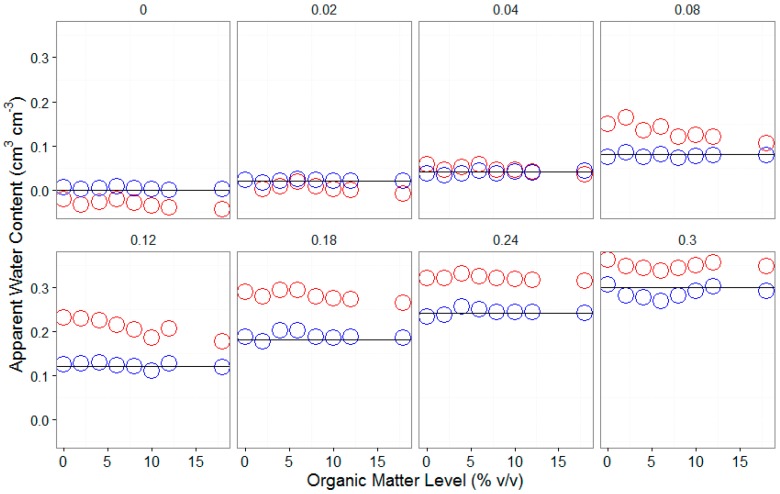
Comparison of apparent water content predicted using the default (red circles) and the new multivariate polynomial calibration (blue circles) equations with actual water content (solid line) as a function of organic matter level.

The organic-matter-level–specific calibration equation improved the accuracy of the tested SWCM sensor (Figure 4 and Figure 5). Using these equations, the accuracy of the sensor varies between 0.013 and 0.019 cm^3^ cm^−3^, while on the other hand, the accuracy of the sensor based on the default calibration equation as determined by the RMSE ranged between 0.053 to 0.072 cm^3^ cm^−3^.

These results concur with those of [13], who reported that low-cost sensors, including the 10HS sensor, lack sensitivity at certain soil water content levels. They further indicated that the accuracy of the 10HS sensor considerably decreased as soils become saturated. This highlights the need for specific calibration equations that account for the soil and environmental conditions. One approach for developing new calibration equations, which takes into consideration the effect of the major physical and chemical properties of soils that could impact the performance of such sensors (e.g., organic matter and salinity), could be achieved by developing multiple calibration equations at different organic matter levels (Table 2 and Figure 4). Organic-matter-level–specific calibration equations (Table 2) fitted the actual water content very well. However, instead of using multiple organic-matter-level-specific calibration equations, developing a multivariate polynomial calibration equation that accounts for multiple variables as covariates could be a very good practical alternative. 

### 3.3. Correcting Effect of Organic Matter on Sensor Readings

Organic matter had a significant effect on the sensor readings (Table 1 and Figure 1). The accuracy and precision of the sensor were significantly affected by the organic matter level. These results concur with those of [32] who reported distinct instrument sensitivity of the 10HS sensor. In contrast, however, Spelman et al. [20] reported that the sensitivity of the 10HS sensor was less under laboratory conditions than under field conditions. Overall, our findings confirm the recommendations by previous studies [13,19,27,33] for the need of a soil-specific calibration equation of SWCM sensors. This study’s findings clearly show that the addition of organic matter results in more water bound to the organic matter particles which are not detected by sensors, and thus, cause an underestimation of sensor readings with an increase in organic matter levels. A study by Rawls et al. [34] reported that the water retention of sandy soils was significantly affected by the addition of organic matter. Although no previous studies investigated the effect of organic matter on sensor readings, several studies have made similar recommendations to account for salinity and temperature effects [8,16,18,27,35,36].

This underscores the need to account for the effect of organic matter on sensor readings. This can be achieved in two ways: (1) by developing organic-matter-level–specific calibration equations as presented in Table 2 and Figure 4; or (2) by developing a multivariate polynomial calibration equation that incorporates the organic matter level as a covariate with raw counts (Equation (3)).

*θ_p_* = 3.252 × 10^−3^ × RC − 3.4 × 10^−6^ × RC^2^ + 1.3 × 10^−9^ × RC^3^ − 1.246 × 10^−2^ × OM + 2.81 × 10^−5^ × OM × RC − 1.3 × 10^−8^ × RC ^2^ × OM + 2.97 × 10^−5^ × OM^2^ − 9.5 × 10^−8^ × RC × OM^2^ + 1.9 × 10^−6^ × OM^3^ –1.053 (3)

where *θ_p_* is the apparent water content (cm^3^ cm^−3^), OM is the organic matter level (%), and RC is the sensor raw counts. 

**Figure 6:**
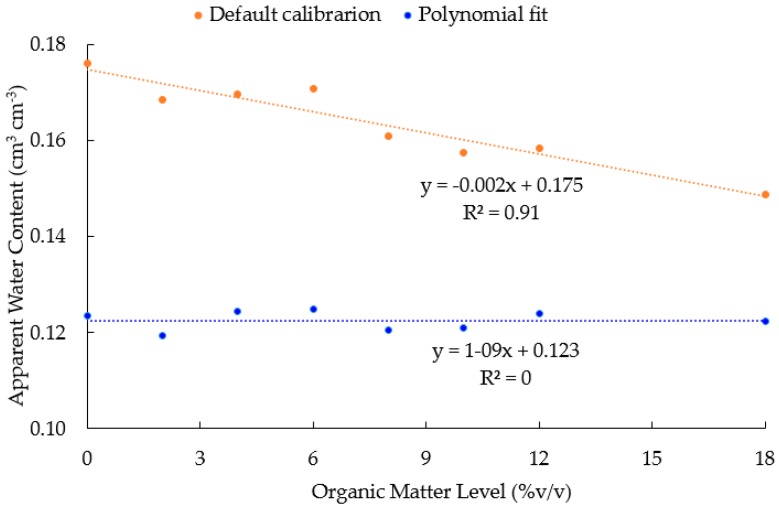
Correlation between organic matter level and apparent water content based on the default calibration (before correction) and the multivariate polynomial calibration equation (after correction).

The new multivariate polynomial calibration equation, with raw counts and the organic matter level as covariates, fitted very well with the actual water content (see “blue” circles in Figure 5) as compared to the default calibration equation (e.g., see “red” circles in Figure 5). Similarly, the negative correlation between the sensor readings and organic matter level (Figure 1) was corrected using the new multivariate calibration equation (Figure 6). 

The multivariate polynomial calibration equation had a residual standard error of 0.014 cm^3^ cm^−3^ and a coefficient of determination (*R*^2^) of 0.98. Overall, the two approaches of developing new calibration equations (organic-matter-level–specific and multivariate polynomial calibration equations) fitted the actual water content better than the default calibration equation provided by the manufacturer (Table 3). The mean absolute error was reduced to 0.01 cm^3^ cm^−3^ for the new calibration equations from 0.054 cm^3^ cm^−3^ for the default, which shows the improvement in the accuracy of the sensor. The absolute error for the default calibration equation (0.054 cm^3^ cm^−3^) of the 10HS sensor was greater than that of the EC-20 sensor under laboratory calibrations (0.012 cm^3^ cm^−3^) observed by Kinzli et al. [37]. 

**Figure 7:**
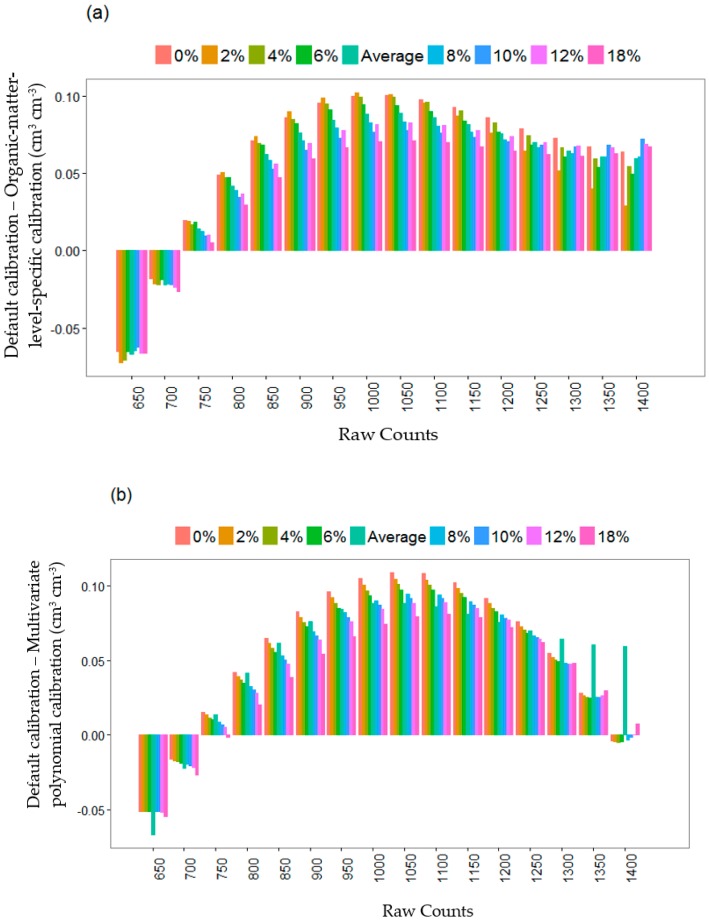
Uncertainties of the default calibration equation compared with organic-matter-level–specific calibration (**a**) and multivariate polynomial calibration equation (**b**).

Moreover, the default calibration equation underestimated the apparent water content by up to 0.054 cm^3^ cm^−3^ at the drier portion of the soil water content range (<750 raw counts) (Figure 7a). In contrast, at the wetter portion of the soil water content range (≥750 raw counts) its predictions were greater than those of the new calibration equations by up to 0.01 cm^3^ cm^−3^. Uncertainties of the default calibration as compared to those of the organic-matter-level–specific and multivariate polynomial calibration equations mostly show similar trends. However, at greater sensor readings (>1100 raw counts), the differences between the default calibration and the organic-matter-level-specific calibration equations were more or less the same, while there was a relatively greater decrease in the differences between the default and the multivariate polynomial calibration equations (Figure 7b). 

## 4. Conclusions

This study is the first scientific experiment that reports on the effects of organic matter on the precision and accuracy of an electromagnetic soil water content monitoring sensor. Different rates of oven-dried sawdust (0%, 2%, 4%, 6%, 8%, 10%, 12% and 18% v/v) were mixed with quartz sand and packed into two-liter glass columns. Sensor readings were recorded at different water content levels by adding appropriate volumes of deionized water to achieve 0, 0.02, 0.04, 0.08 0.12, 0.18, 0.24 and 0.30 cm^3^ cm^−3^. Organic matter had a significant effect (*p* < 0.05) on the precision and accuracy of the tested sensor, resulting in an underestimation of the soil water content at the dry water content range (<0.05 cm^3^ cm^−3^) while overestimating the soil water content at the relatively higher water content range (>0.05 cm^3^ cm^−3^). This confirmed the need for correcting the organic matter effect on the performance of SWCM sensors. The water content estimated by the new calibration equations, organic-matter-level–specific calibration and multivariate polynomial calibration, which accounts for the organic matter level, better fitted the actual water content in the sand columns compared to the water content estimated by the default calibration equation. Overall, the mean absolute error was reduced to 0.01 cm^3^ cm^−3^ for the new calibration equations from 0.054 cm^3^ cm^−3^ by the default calibration equation. 

The findings of this study suggest that the addition of organic matter to quartz sand results in more water bound to organic particles that become less accessible to SWCM sensors, and thus leads to an underestimation of the sensor readings. Overall, our findings confirmed that organic matter significantly affects sensor readings and thus there is a need for further investigations on the effect of organic matter on the performance of different SWCM sensors under different soil types and environmental conditions. 

## Figures and Tables

**Table 1:** Analysis of variance of sensor readings (RC) as a function of organic matter level (OM), sensor type (ST), and water content (WC).

Source of Variation	*df*	Sum of Squares	Mean Squares	F	*p*-Value
OM	7	91,523.4	13,075.0	17,038.0	<0.001
ST	2	101,709.0	50,854.0	66,269.5	<0.001
WC	7	27,650,000.0	3,950,152.0	5,147,527.0	<0.001
OM × ST	14	6782.9	484.0	631.4	<0.001
OM × WC	49	107,498.0	2194.0	2858.8	<0.001
ST × WC	14	30,686.4	2192.0	2856.3	<0.001
OM × ST × WC	98	48,748.0	497.0	648.2	<0.001
Error	382	293.1	1		
Total	575	28,040,000.0			

**Table 2:** Summary of organic-matter-level–specific calibration equations (*n* = 72).

Organic Matter Level (% v/v)	Organic-Matter-Level-Specific Calibration Equation			Organic-Matter-Level-Specific Calibration		Default Calibration
	(×10^−7^)	(×10^−4^)	(×10^−2^)	*R*²	RMSE (cm^3^ cm^−3^)	RMSE (cm^3^ cm^−3^)
0	3.67 × RC^2^ − 2.69 × RC + 2			0.98	0.015	0.072
2	5.26 × RC^2^ − 5.60 × RC + 15			0.98	0.013	0.070
4	4.14 × RC^2^ − 3.60 × RC + 7			0.97	0.019	0.070
6	3.74 × RC^2^ − 2.64 × RC + 2			0.96	0.019	0.067
8	2.54 × RC^2^ − 0.32 × RC − 8			0.98	0.016	0.060
10	1.63 × RC^2^ + 1.41 × RC − 16			0.98	0.014	0.057
12	2.24 × RC^2^ + 0.15 × RC − 10			0.98	0.014	0.061
18	1.51 × RC^2^ + 1.68 × RC − 17			0.99	0.013	0.053

**Table 3:** Comparisons between accuracy of the default calibration on one hand and the organic-matter-level–specific and multivariate polynomial calibration equations on the other hand.

Mean Absolute Error (MAE, cm^3^ cm^−3^)			
Organic Matter Level (% v/v)	Default Calibration	Organic-Matter-Level-Specific Calibration Equation (RC)	Multivariate Polynomial Equation (RC and OM)
0	0.059	0.012	0.011
2	0.060	0.010	0.010
4	0.057	0.015	0.011
6	0.055	0.014	0.012
8	0.049	0.012	0.010
10	0.049	0.010	0.009
12	0.053	0.011	0.010
18	0.047	0.010	0.008
